# Supplementary material for: Search for Ancestral Features in Genomes of Rhizobium leguminosarum bv. viciae Strains Isolated from the Relict Legume Vavilovia formosa
Source: Genes (Basel). 2019 Dec 1;10(12):990. doi: 10.3390/genes10120990 (PMC6969944; doi:10.3390/genes10120990)
Supplement: Supplementary file 1 [file genes-10-00990-s001.pdf]

Table S1. Average nucleotide identity of genomes of *Rhizobium leguminosarum* bv. *viciae* strains.

	**248**	**3841**	**TOM**	**Vaf-10**	**Vaf-108**	**Vaf-12**	**WSM1481**
**248**	100,00%						
**3841**	94,38%	100,00%					
**TOM**	94,66%	94,24%	100,00%				
**Vaf-10**	94,63%	94,28%	95,34%	100,00%			
**Vaf-108**	94,99%	94,30%	95,36%	96,26%	100,00%		
**Vaf-12**	94,44%	95,10%	94,56%	94,95%	95,11%	100,00%	
**WSM1481**	94,31%	95,85%	94,22%	94,25%	94,28%	95,08%	100,00%

Table S2. Average nucleotide identity of chromosomes of *Rhizobium leguminosarum* bv. *viciae* strains.

	**248**	**3841**	**TOM**	**Vaf-10**	**Vaf-12**	**Vaf-108**	**WSM1481**
248	100,00%						
3841	94,47%	100,00%					
TOM	94,89%	94,42%	100,00%				
Vaf-10	94,89%	94,50%	95,69%	100,00%			
Vaf-12	94,65%	95,30%	94,80%	94,92%	100,00%		
Vaf-108	95,23%	94,51%	95,71%	95,93%	95,06%	100,00%	
WSM1481	94,53%	95,90%	94,46%	94,49%	95,30%	94,52%	100,00%

Table S3. Core and accessory genome statistics.

**Strain**	**All strains**	**Vavilovia's strains**	**Derived group**	**Derived group excluding TOM**
Core genes (99% <= strains <= 100%)	3817	4607	3978	4167
Soft core genes (95% <= strains < 99%)	0	0	0	0
Shell genes (15% <= strains < 95%)	4897	2892	2810	1890
Cloud genes (0% <= strains < 15%)	1204	0	0	0
Total genes (0% <= strains <= 100%)	9918	7499	6788	6057

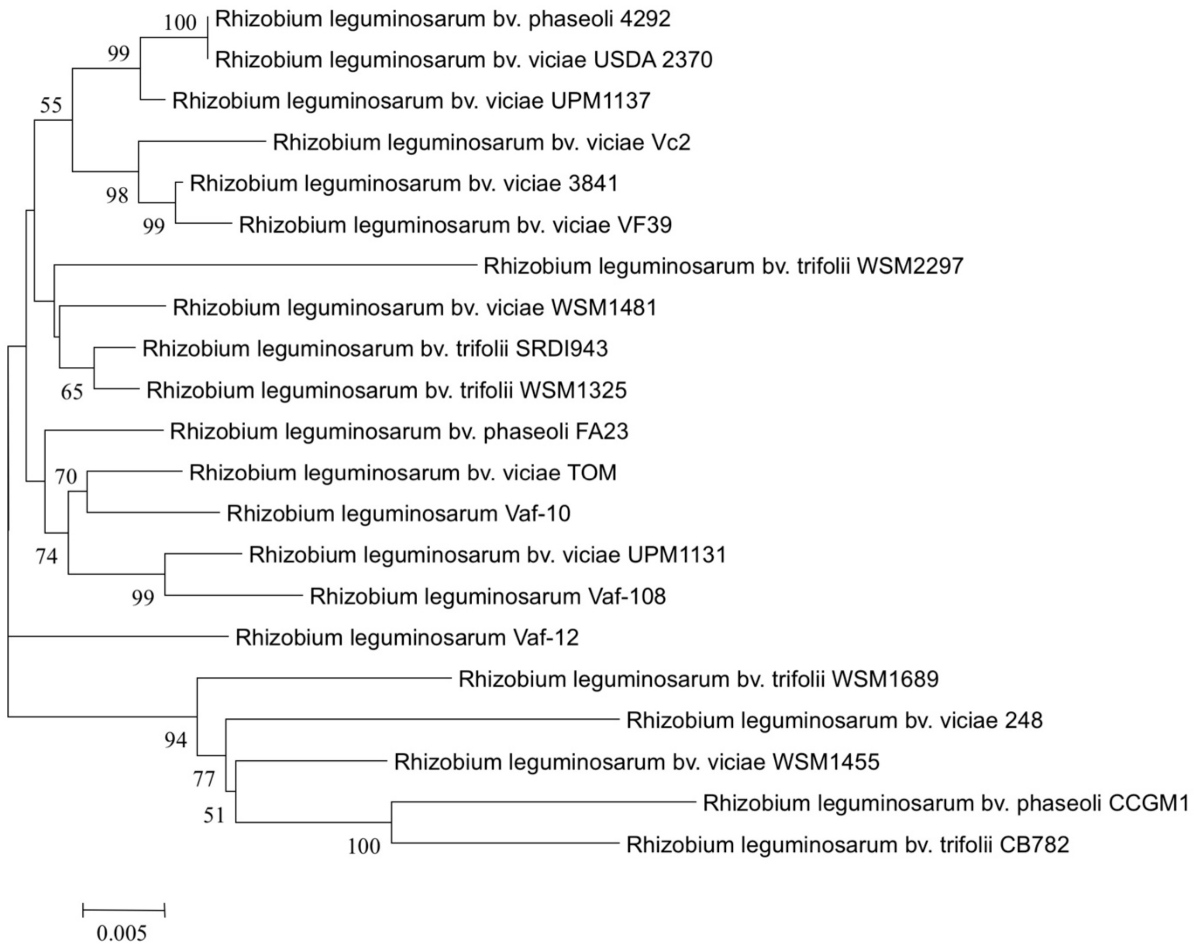

Figure S1. Neighbor joining tree for concatenate of core genes (*16S rRNA*, *dnaK*, *glnA* and *gsII*). The evolutionary distances were computed using the maximum composite likelihood method. Values of bootstrap test (1000 replicates) exceeding 50 are shown next to the branches. [19]

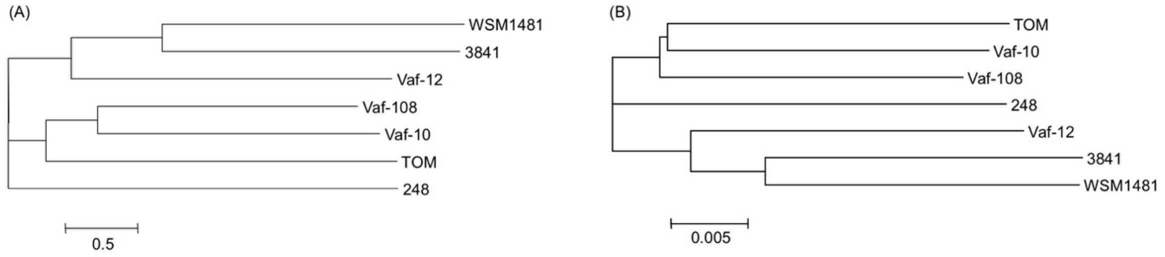

Figure S2. ANI-distance clustering. (A) Whole genomes. (B) Chromosomes.

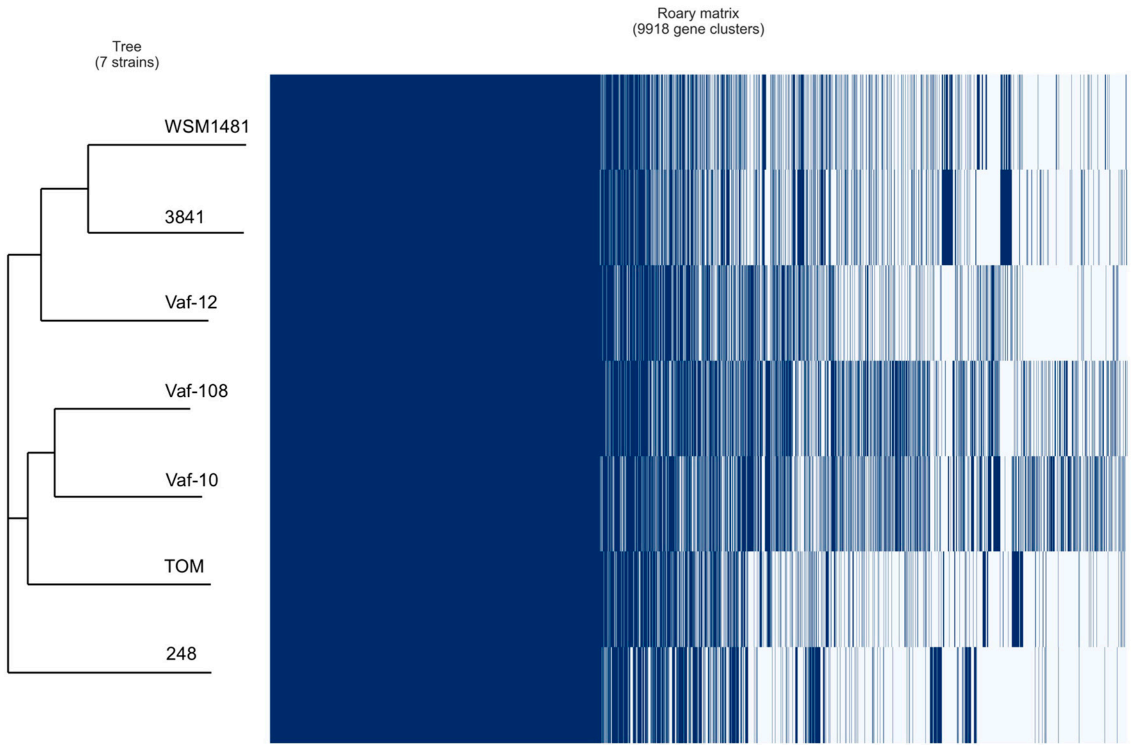

Figure S3. The matrix with the presence and absence of core and accessory genes.in comparison with phylogram of ANI-distance clustering (Figure S1A).

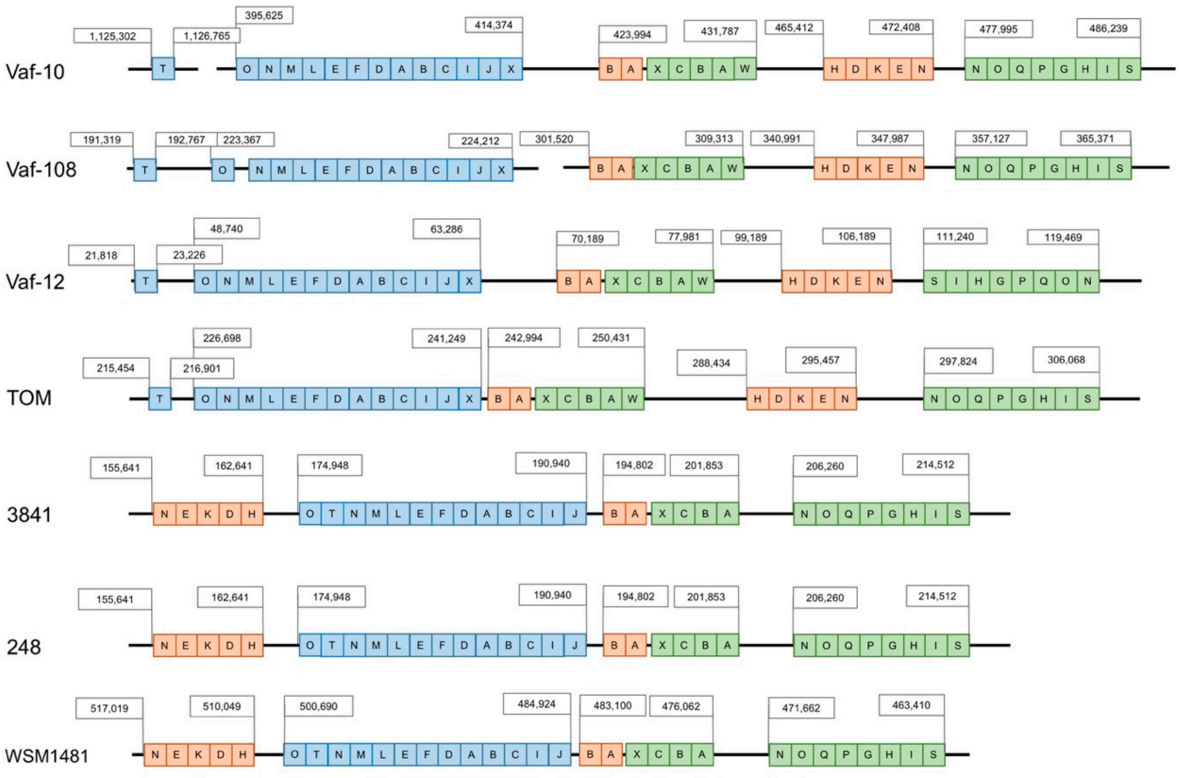

Figure S4. Schematic structure of *R. leguminosarum* Sym regions. Blue, *nod* genes; orange, *nif* genes; green, *fix* genes. Nucleotide positions are labeled with flags. Accession numbers of sequences are: Vaf-10, CP016287 (*nod*T), CP016290 (other *sym* genes); Vaf-108, CP018235 (*nod* genes), CP018229 (*nif* and *fix* genes); Vaf-12, KT944070; TOM, AQUC01000005; 3841, NC_008381; 248, ARRT01000005; WSM1481, AQUM01000002.
